# Successful thoracoscopic-assisted explant of a notably migrated aortic stent graft protruding into the posterior mediastinum after endovascular re-exclusion

**DOI:** 10.1016/j.jvscit.2026.102244

**Published:** 2026-03-31

**Authors:** Yumi Kakizawa, Yoshiki Watanabe, Kazuo Shimamura, Kazuma Handa, Koichi Maeda, Takashi Shirakawa, Satoshi Sakakibara, Fumio Yamana, Saito Shunsuke, Daisuke Yoshioka, Shigeru Miyagawa

**Affiliations:** Department of Cardiovascular Surgery, The University of Osaka, Graduate School of Medicine, Osaka, Japan

**Keywords:** Thoracic endovascular aortic repair, Stent graft migration, Removal of stent graft, Minimally invasive surgery, Thoracoscopy-assisted minithoracostomy

## Abstract

We report a case of an 88-year-old patient in whom an aortic stent graft was placed in the distal aortic arch, which showed structural disintegration with excessive distal migration 16 years after implantation. Eventually, the graft penetrated the descending aortic wall and protruded into the posterior mediastinum. The patient was treated successfully without open conversion using a staged minimally invasive approach, which included repeat stent graft placement in the aortic arch and removal of the migrated stent graft via a thoracoscopy-assisted right minithoracostomy.

Thoracic endovascular aortic repair (TEVAR) has been widely accepted as an effective and less invasive treatment option for various aortic pathologies. Nevertheless, late complications, such as endoleaks and stent graft migration, remain a concern in long-term follow-up. Here, we report a rare case of severe distal migration of a stent graft placed in the distal aortic arch that led to aortic wall penetration and protrusion into the posterior mediastinum more than a decade after the initial procedure. Considering the patient's advanced age, the patient was successfully treated using a staged minimally invasive approach tailored to minimize invasiveness and avoid open conversion. Written informed consent for publication of this case was obtained from the patient.

## Case report

**Fig 1:**
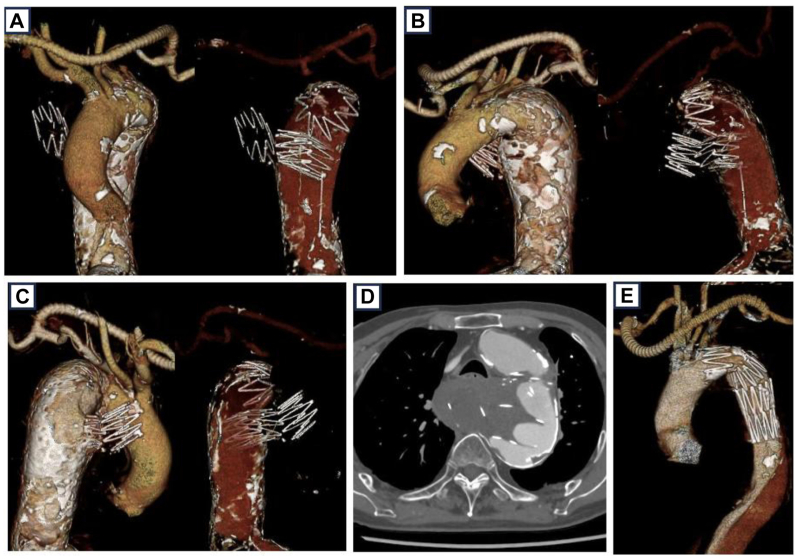
Computed tomography (CT) findings before and after stent graft migration. **(A-C)** Three-dimensional reconstructed CT images showing the thoracic aorta from different angles. The *right panels* in each pair depict the same views with the stent graft struts rendered visible to demonstrate the configuration and marked distal migration of the fractured components protruding into the posterior mediastinum. **(D)** Axial contrast-enhanced CT image demonstrating the migrated stent graft within the posterior mediastinum, compressing the esophagus. **(E)** Three-dimensional CT image obtained immediately after the initial debranching thoracic endovascular aortic repair (TEVAR) performed 16 years earlier, showing the intact custom-made stent graft in the distal aortic arch.

An 88-year-old man with dysphagia and hoarseness was referred to our hospital after a computed tomography (CT) scan revealed a migrated stent graft in the posterior mediastinum. Sixteen years earlier, he had undergone zone 2 TEVAR with supra-aortic debranching, including a right-to-left axillary artery bypass and custom stent grafts made of woven polyester and stainless-steel Z-stents, for a dissected aortic aneurysm that had enlarged to a maximum diameter of 60 × 56 mm. Custom stent grafts composed of woven polyester and stainless steel Z-stents were used for the procedure and constructed by securing all Z-stent apices to the graft with interrupted 5-0 Prolene sutures. The proximal landing zone was a nondissected segment measuring 29.0 mm, and the distal landing zone was the true lumen measuring 21.8 mm, with an overall aortic diameter of 38.9 mm at that level. A tapered stent graft (33 mm proximally and 27 mm distally) was used. A postoperative CT scan demonstrated expansion of the distal true lumen to 22.8 mm without evidence of intimal disruption, with good apposition to the true lumen wall. The patient underwent regular follow-up with clinical assessment and CT imaging approximately every 6 months for the first 3 years and annually for the subsequent 5 years, during which no migration or other complications were observed. However, routine surveillance was discontinued after the patient was transferred to a hospital near his home, and no interval imaging was available for the following 8 years until the current presentation. A contrast-enhanced CT scan revealed disintegration of the four Z-stents, with one remaining in the aortic arch and the other three migrating distally. The migrated components penetrated the aortic wall and protruded into the posterior mediastinum, resulting in pseudoaneurysm formation. The migrated stents and pseudoaneurysm appeared to compress the esophagus dorsally, contributing to dysphagia (Fig 1).

To prevent rupture, aneurysm excision or exclusion was deemed necessary, and removal of the stent graft was considered essential to alleviate dysphagia and prevent future esophageal injury. Given his advanced age and limited physiological reserve, he was considered unfit for open chest graft replacement. Although total excision of the pseudoaneurysm was considered, dense mediastinal adhesions were anticipated. Therefore, a safer, less invasive staged strategy was planned, consisting of additional stent graft placement followed by removal of the migrated graft via a right minithoracostomy.

### First stage procedure: Zone 1 TEVAR

To ensure adequate proximal sealing, TEVAR extending to zone 1 was deemed necessary. Because a right-to-left axillary artery bypass had already been created during the initial procedure, an additional bypass from the left common carotid to the left subclavian artery was performed via a supraclavicular approach.

**Fig 2:**
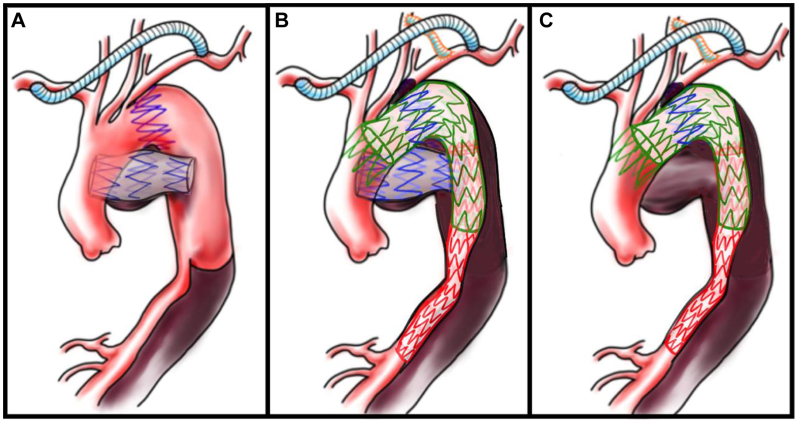
Schematic illustration of staged hybrid management. **(A)** Preoperative schema showing the relationship between the aorta and the migrated stent graft. Of the four serial stent segments from the initial custom-made graft, three (*blue*) had migrated distally and penetrated the posterior mediastinum, forming a pseudoaneurysm. **(B)** After the additional debranching thoracic endovascular aortic repair (TEVAR). After the left common carotid-left axillary artery bypass, a Relay Pro (*green*) and a conformable TAG (*red*) stent graft were deployed within the true lumen of the descending aorta to exclude the pseudoaneurysm. **(C)** After thoracoscopic-assisted mini-right thoracostomy and removal of the migrated stent graft. The proximal Z-stent from the original graft remained outside the Relay Pro stent graft within the distal arch and was left in place without complications.

A 20F DrySeal sheath (W. L. Gore & Associates) was inserted through the external iliac artery, and a conformable TAG (28-28-200 mm; W. L. Gore & Associates, Flagstaff) was deployed from the distal arch to the mid-descending thoracic aorta, covering and extending beyond the migrated stent graft. During this step, the guidewire was carefully advanced through the appropriate true lumen pathway to avoid interaction with the migrated graft. Subsequently, a Relay Pro (36-32-190 mm; Terumo Aortic) was deployed just distal to the brachiocephalic artery to complete the proximal seal (Fig 2). The postoperative course was uneventful, and a follow-up CT scan on postoperative day 6 showed no endoleaks.

### Second stage procedure: Removal of stent graft with thoracoscopic-assisted right minithoracostomy

**Fig 3:**
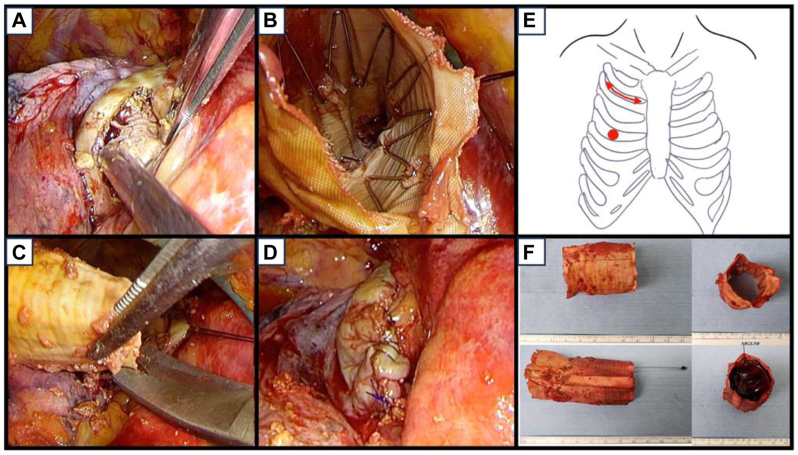
Intraoperative thoracoscopic-assisted minithoracotomy findings and schematic illustration. **(A)** Opening of the pseudoaneurysm revealed old atheromatous debris and organized white thrombus without active bleeding. The migrated stent graft was identified deep within the cavity. **(B)** Intraluminal view of the extracted stent graft, showing metallic stent struts and organized thrombus. **(C)** The graft was divided into three segments and removed in portions; the first exposed section contained no stent component. **(D)** The opened pseudoaneurysm wall was closed with continuous 3-0 Prolene sutures. **(E)** Schematic representation showing the operative approach: a right minithoracotomy through the second intercostal space with thoracoscopic assistance via the fourth intercostal space, enabling a minimally invasive procedure. **(F)** Gross photographs of the retrieved stent graft segments. The later-extracted segment was filled with red thrombus.

On postoperative day 8, under general anesthesia with single-lung ventilation, a right minithoracostomy was performed through the second intercostal space with thoracoscopic assistance using a camera port at the fourth intercostal space. After dissecting lung adhesions, the pseudoaneurysm was exposed and incised. An old thrombus was found within the aneurysmal sac and removed. The graft portion without the stent was first identified and carefully extracted from the pseudoaneurysm, revealing that the proximal side of the stent graft had dislodged into the posterior mediastinum and was no longer connected to the remaining Z-stent in the distal arch. After excising the proximal portion of the graft, the second through fourth migrated sections of the stent graft were successfully removed from the aneurysmal cavity without significant bleeding. The first Z-stent alone, without the graft component, was left in the distal arch and considered harmless because it remained outside the Relay stent graft. Finally, the aneurysm wall was closed using sutures (Fig 3).

**Fig 4:**
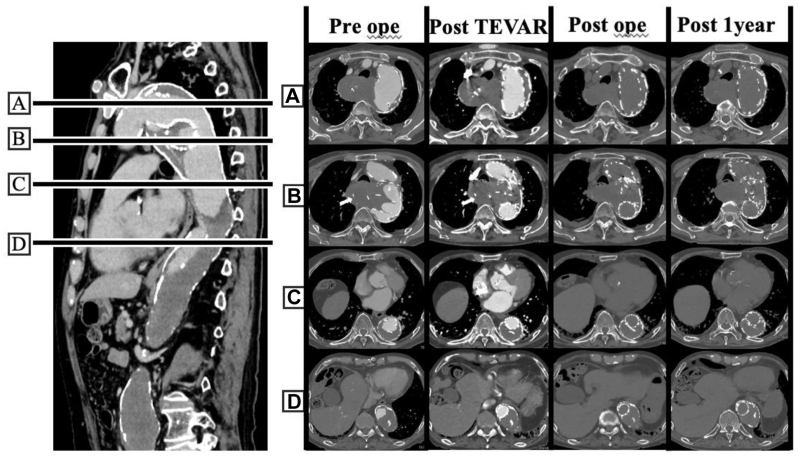
Preoperative oblique view along the aorta and serial axial computed tomography (CT) images at each time point. Preoperative oblique CT image along the aorta (*left*) and serial axial CT scans at four representative levels (**A**, distal arch; **B**, migrated stent graft; **C**, aortic valve; **D**, diaphragm) before surgery, after zone 1 thoracic endovascular aortic repair (TEVAR), after stent graft removal, and at 1-year follow-up. After zone 1 TEVAR, the blood flow within the aorta was completely excluded from the migrated stent graft without any endoleak. Postoperative CT scan, obtained without contrast because of concern for renal dysfunction, confirmed complete removal of the migrated stent graft. At 1 year, the pseudoaneurysm had neither enlarged nor regressed, indicating a stable condition.

The patient's postoperative course was uneventful. He was discharged walking independently 1 month after rehabilitation. His dysphagia resolved immediately after the second-stage procedure, and his hoarseness completely improved 1 month postoperatively. A follow-up CT scan at 1 year showed no reduction or enlargement the pseudoaneurysm, indicating lesion stability (Fig 4).

## Discussion

Stent graft migration is a known long-term complication of TEVAR, typically defined as displacement of >10 mm.^1^ It has been reported to occur in 1% to 3% of cases undergoing TEVAR.^2^^,^^3^ Migration in the chronic phase may result from structural failure or aortic morphological changes.^4^^,^^5^ In the present case, a stent graft fracture was confirmed, and the graft protruded into the posterior mediastinum, representing a rare and unusual type of migration. Follow-up imaging obtained 8 years after the initial procedure demonstrated progressive enlargement of the distal true lumen, with expansion of the distal stent graft diameter from 22.8 mm in the early postoperative period to 29.0 mm. Although no obvious structural disruption of the aortic wall, such as stent-induced new entry, was observed, this morphological change may have decreased the radial force of the stent graft and its mechanical apposition to the native aortic wall, potentially leading to gradual instability and distal migration.

However, because only noncontrast CT was available during this period owing to renal dysfunction, the presence of an endoleak could not be definitively assessed. Therefore, the exact mechanism of migration remains uncertain and was likely multifactorial, involving both structural deterioration of the custom-made stent graft and progressive aortic morphological change.

In general, the treatment of stent graft migration involves additional stent graft placement to control endoleaks or reinforce anatomical fixation.^6^ However, when the migrated stent graft causes significant anatomical distortion, obstructs subsequent treatment, or directly threatens the surrounding tissues, surgical removal is often required. Open conversion with graft explantation and aortic reconstruction are necessary in such situations.^7^^,^^8^ In this case, graft removal was essential not only to facilitate retreatment but also to prevent further tissue damage caused by the migrating stent graft.

Nevertheless, considering the patient's advanced age and frailty, conventional open repair via an extended thoracotomy or clamshell incision was deemed intolerable. Therefore, we planned a two-stage hybrid strategy consisting of additional endovascular repair to seal the aorta, followed by minimally invasive graft removal through a thoracoscopic-assisted minithoracostomy. This approach represents a novel treatment paradigm for complex cases.

Several important considerations were required to safely perform this procedure. First, complete endoleak control was essential during the first-stage TEVAR, because opening the aneurysmal sac during the second-stage procedure could lead to bleeding if any residual endoleak remained. To achieve secure sealing, a conformable TAG was selected for the distal segment because its spiral Z-stent configuration was considered more suitable for the narrowed, elliptical true lumen. For the proximal segment, a Relay Pro device was chosen to allow more precise deployment just distal to the brachiocephalic artery and because its lower profile delivery system was more appropriate for the relatively small access route. A postoperative contrast-enhanced CT scan was performed after the first stage to confirm the absence of endoleaks, including type II. Additionally, we prepared for potential intraoperative bleeding during the second stage by ensuring the availability of fluoroscopic guidance and an aortic occlusion balloon that could be advanced proximal to the newly implanted stent graft.

Second, the wire pathway must be meticulously planned during TEVAR. In this case, improper wire passage could have pushed the migrated stent graft deeper into the posterior mediastinum during deployment of the new stent graft. To avoid this complication, the wire was deliberately advanced along the anterior side of the migrated graft to minimize interaction. Similarly, in the distal aortic arch, we ensured that the wire passed through the center of the residual Z-stent frame to allow symmetric expansion of the new stent graft and prevent entanglement. Consequently, the remaining stent frame was gently displaced outward within the vessel wall without excessive tension, minimizing the risk of future complications.

Each case may present unique anatomical relationships; therefore, thorough preoperative assessment is essential to predict how the migrated stent graft will respond to new graft deployment. Strategic planning tailored to each patient's anatomy is crucial for the success of this minimally invasive recovery approach. This report has several limitations. It was difficult to distinguish whether the protruding sac represented a pseudoaneurysm or an outwardly protruding false lumen based on CT findings alone, and histopathological evaluation of the sac wall was not performed. Therefore, the exact nature of the cavity could not be definitively determined. However, given that false lumen protrusion across the mediastinum without wall disruption is unlikely, the lesion was described as a pseudoaneurysm based on anatomical considerations. Furthermore, the reason why rupture did not occur before the patient's presentation remains unclear.

## Conclusions

We encountered a case in which a previously implanted stent graft migrated to a rare position in the posterior mediastinum. The stent graft was successfully removed using a two-stage hybrid procedure, including zone 1 TEVAR and thoracoscopy-assisted right minithoracostomy.

## Funding

None.

## Disclosures

None.
